# Supplementary material for: Zinc isotopic evidence for recycled carbonate in the deep mantle
Source: Nat Commun. 2022 Oct 14;13:6085. doi: 10.1038/s41467-022-33789-6 (PMC9568527; doi:10.1038/s41467-022-33789-6)
Supplement: Supplementary file 1 — Supplementary Information [file 41467_2022_33789_MOESM1_ESM.pdf]

Supporting Information for:

Zinc isotopic evidence for recycled carbonate in the deep mantle

Xiao-Yu Zhang[1], Li-Hui Chen[2,1,*], Xiao-Jun Wang[2], Takeshi Hanyu[3], Albrecht W. Hofmann[4], Tsuyoshi Komiya[5], Kentaro Nakamura[6], Yasuhiro Kato[6], Gang Zeng[1], Wen-Xian Gou[1], Wei-Qiang Li[1]

[1] School of Earth Sciences and Engineering, State Key Laboratory for Mineral Deposits Research, Nanjing University, Nanjing 210023, China

[2] Department of Geology, State Key Laboratory of Continental Dynamics, Northwest University, Xi'an 710069, China

[3] Research Institute for Marine Geodynamics, Japan Agency for Marine-Earth Science and Technology, Yokosuka 237-0061, Japan

[4] Abteilung Klimageochemie, Max-Planck-Institut für Chemie, D-55128 Mainz, Germany

[5] Department of Earth Science and Astronomy, The University of Tokyo, Japan

[6] Department of Systems Innovation, School of Engineering, The University of Tokyo, Bunkyo-ku, Tokyo 113-8656, Japan

[*] **To whom correspondence should be addressed:**

Li-Hui Chen; E-mail address: chenlh@nwu.edu.cn or chenlh@nju.edu.cn

Contents:

Supplementary Figures (**Supplementary Figure 1~8**)

Supplementary Tables (**Supplementary Table 1~7**)

Note1: Description for ocean island basalt samples in this study

Note2: Description for ancient carbonates and altered oceanic crust samples in this study

Note3: Description for quantitative modeling of zinc isotopic variations by crystal fractionation

Note4: Description for quantitative modeling of partial melting of different mantle lithologies

Note5: Description for quantitative modeling of mass balance for zinc isotopes

Note6: Data source for Figure 1, Figure 3 and Figure 4

Supplementary Figures (1~8)

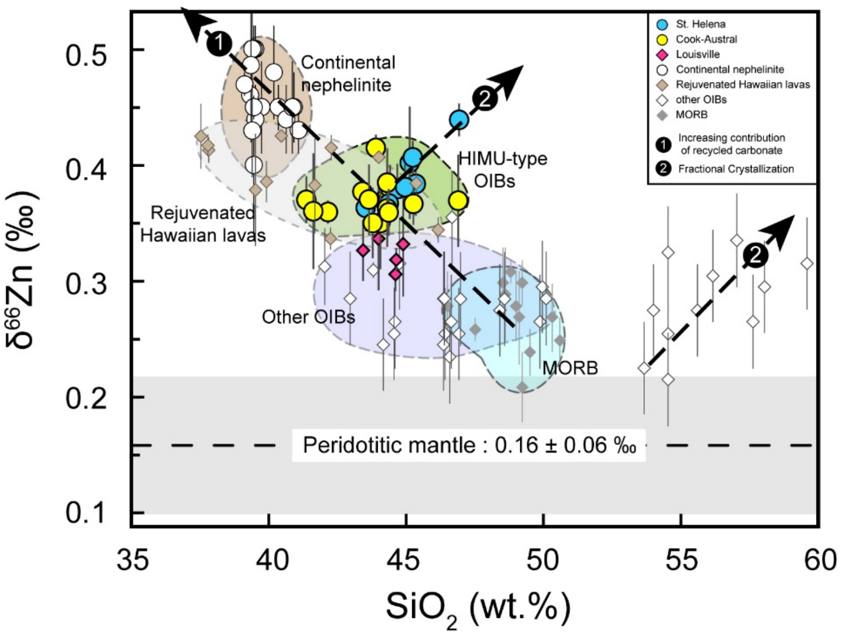

Supplementary Figure 1. Variations in δ^{66}Zn versus SiO_2 for HIMU basalts. Estimated δ^{66}Zn values of peridotitic mantle are taken from ref.[1]. MgO contents for OIB (ocean-island-basalt) samples from Cook-Austral Islands are from refs.[2,3]. Data for Louisville can be found in ref.[4,5]. Literature Data for OIBs are from refs.[6-8]. Data for MORB (mid-ocean ridge basalt) can be found in refs.[6,9,10]. Data for continental nephelinite can be found in refs.[11,12]. Error bars on δ^{66}Zn represent 2 standard deviations (2SD) uncertainties.

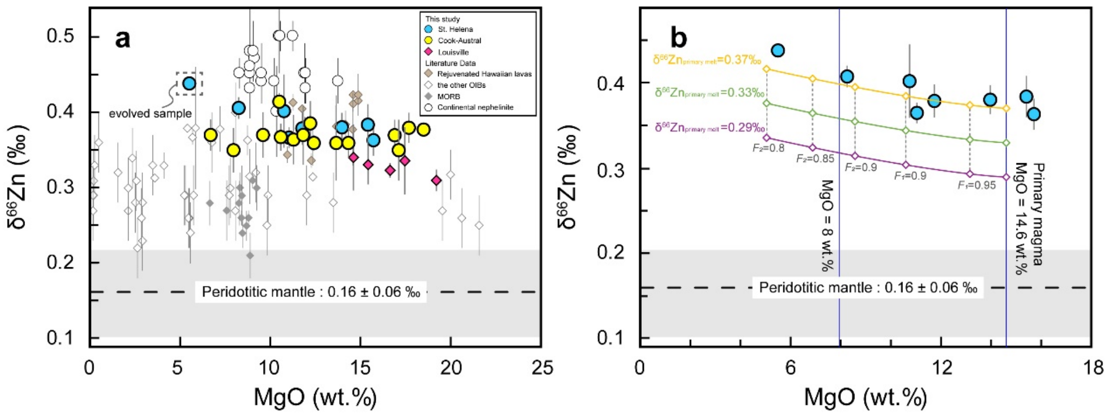

Supplementary Figure 2. (a)Variations in δ^{66}Zn versus MgO for OIB samples in this study. MgO contents for OIB samples from Cook-Austral Islands are from refs.[2,3]. Data for Louisville can be found in ref.[4,5]. Literature Data for OIBs are from refs.[6-8]. Data for MORB can be found in refs.[6,9,10]. Data for continental nephelinite can be found in refs.[11,12]. Error bars on δ^{66}Zn represent 2 standard deviations (2SD) uncertainties. δ^{66}Zn value of peridotitic mantle is given by ref.[1]. The black dashed line marks the average value and the gray field highlights the uncertainty range. **(b) Modeled variations of δ^{66}Zn values during crystal fractionation.** The yellow lines represent the modeling results for crystal fractionation of melt with an initial δ^{66}Zn of 0.37 ‰. The green lines represent the modeling results for crystal fractionation of melt with initial δ^{66}Zn of 0.33 ‰. The purple lines represent the modeling results for crystal fractionation of melt with initial δ^{66}Zn of 0.29 ‰. Detailed description for calculation of crystal fractionation can be found in Supplementary Information, Note 3 and Supplementary Table 3.

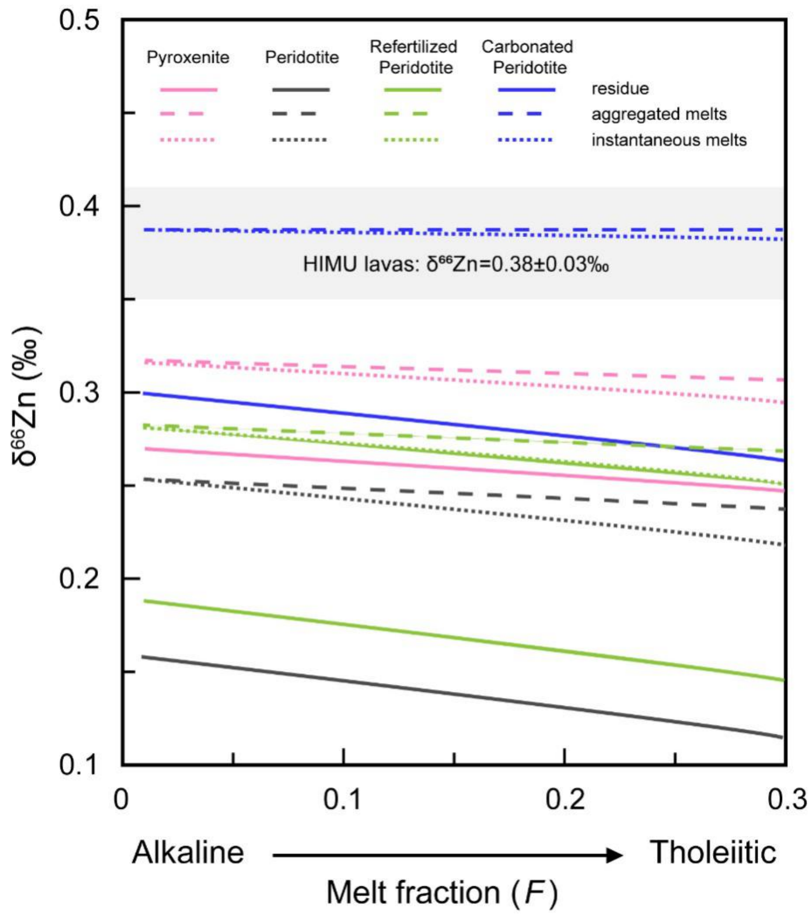

Supplementary Figure 3. Modeled δ^{66}Zn values for melt and residue generated by different degrees of fractional melting of pyroxenite, peridotite, refertilized peridotite and carbonated peridotite. The gray lines represent the modeling results for partial melting of peridotite with an initial δ^{66}Zn of 0.16 ‰. The pink lines represent the modeling results for partial melting of pyroxenite with an initial δ^{66}Zn of 0.27 ‰. The green lines represent the modeling results for partial melting of refertilized peridotite with initial δ^{66}Zn of 0.19 ‰. The blue lines represent the modeling results for partial melting of carbonated peridotite with initial δ^{66}Zn of 0.30 ‰. See Supplementary Table 4 and Supplementary Table 5 for details.

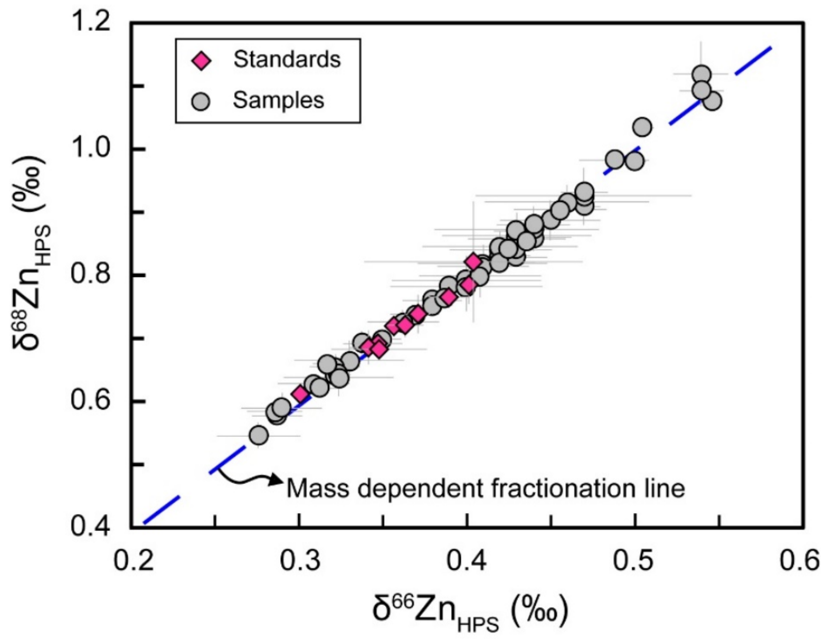

Supplementary Figure 4. Three zinc isotopes diagram for samples and standards analyzed in this study. All data in this study fall on the mass-dependent fractionation line. All error bars represent 2 standard deviations (2SD) uncertainties.

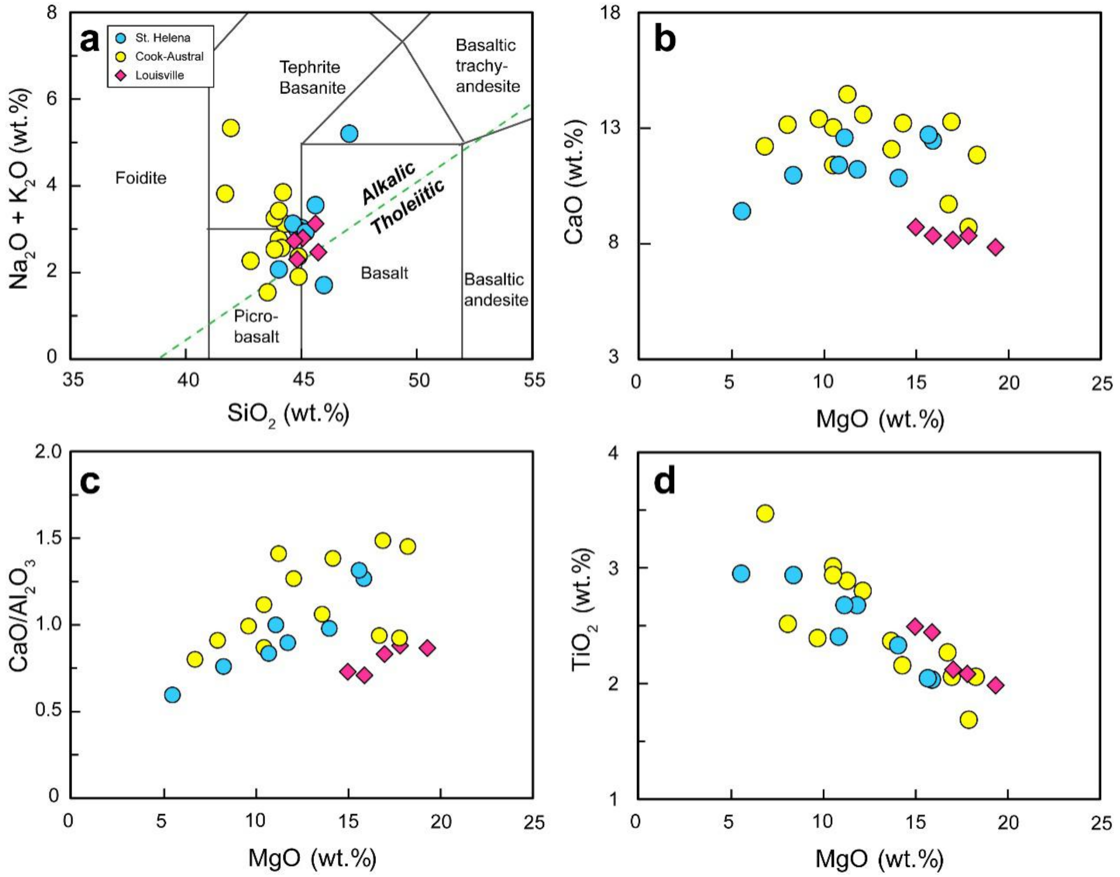

Supplementary Figure 5. Major element variations for OIB samples in this study. (a) Variations in total alkali (Na_2O+K_2O) versus SiO_2 contents for samples. (b) Variations in CaO versus MgO contents for samples. (c) Variations in CaO/Al_2O_3 ratios versus MgO contents for samples. (d) Variations in TiO_2 versus MgO contents for samples. Data for Cook-Austral samples are from refs. [2,3]. Data for St. Helena samples are given by ref. [13]. Data for Louisville samples can be found in ref. [4,5]. All data in this figure are given in Supplementary Table 7.

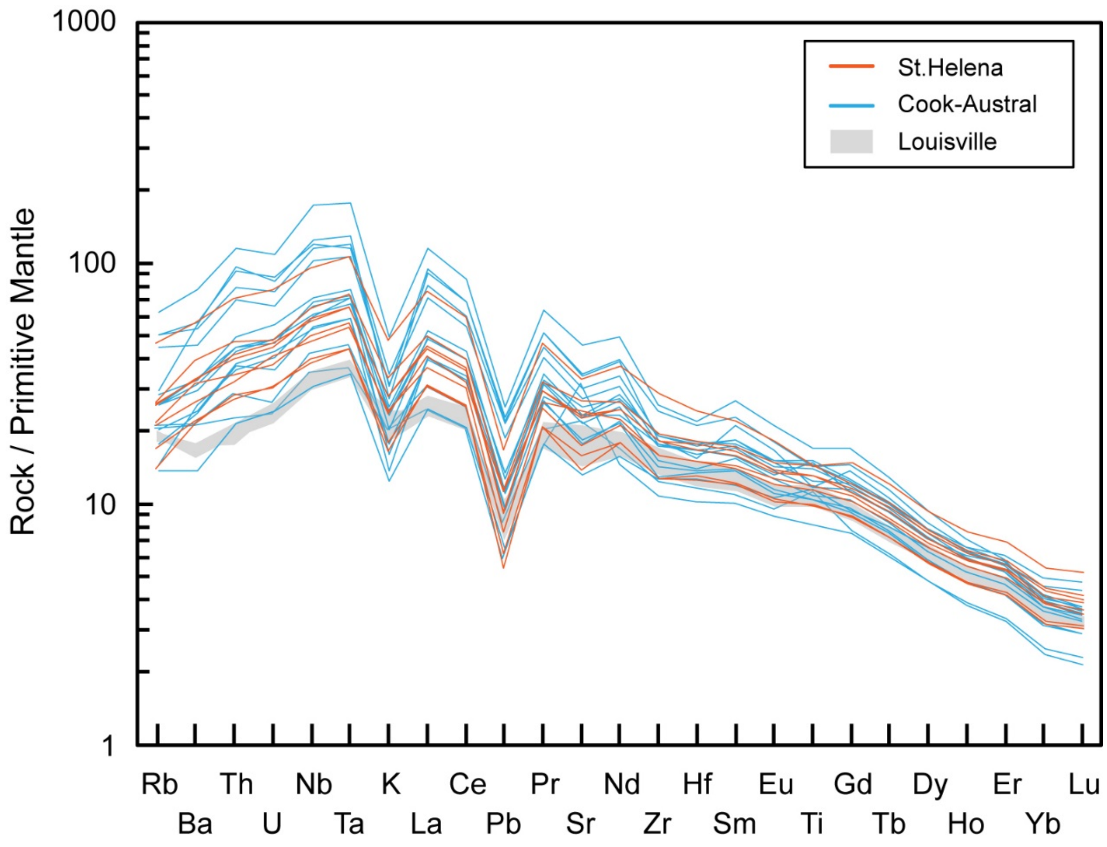

Supplementary Figure 6. Primitive-mantle-normalized incompatible element diagram. Data for Cook-Austral Islands are from ref.[2,3]. Data for St. Helena Island are given by ref.[13]. Data for Louisville can be found in ref.[4,5]. All data in this figure are given in Supplementary Table 7.

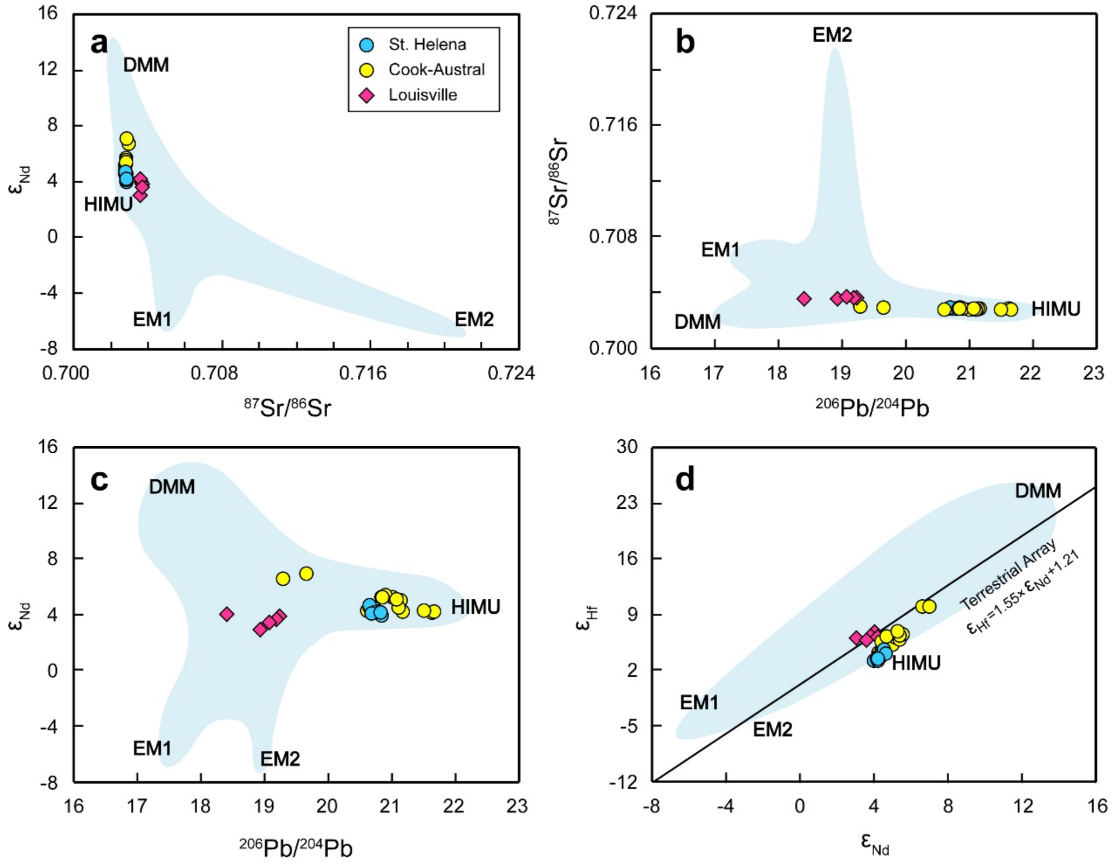

Supplementary Figure 7. Sr, Nd, Pb, and Hf isotopic compositions of OIB samples in this study. (a) The plot of ε_{Nd} values versus $^{87}Sr/^{86}Sr$ ratios. (b) The plot of $^{87}Sr/^{86}Sr$ versus $^{206}Pb/^{204}$ Pb ratios. (c) The plot of ε_{Nd} values versus $^{206}Pb/^{204}$ Pb ratios. (d) The plot of ε_{Hf} versus ε_{Nd} values. Data for Cook-Austral Islands are from ref.[2,3]. Data for St.Helena Island are given by ref.[13]. Data for Louisville can be found in ref.[4,5]. The equation for terrestrial array in panel (d) is from ref.[14]. Background data are from the GEOROC database (http://georoc.mpch-mainz.gwdg.de/georoc).

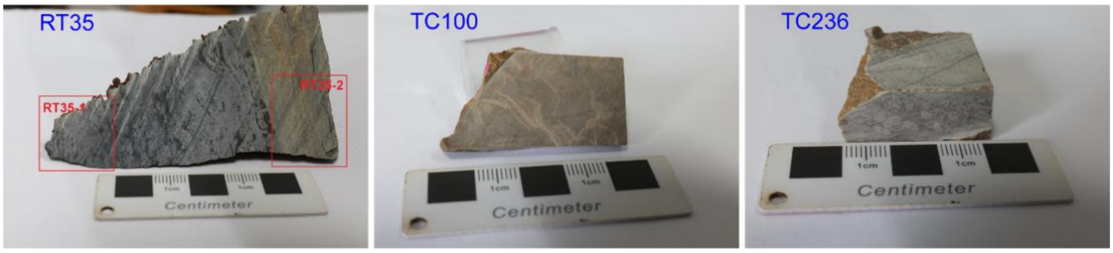

Supplementary Figure 8. Photographs for one Archean carbonate sample (RT35) and two early Proterozoic carbonate samples (TC100 and TC236) in this study. We cut two pieces (RT35-1, RT35-2) from RT35 for Zn isotopic analysis.

Supplementary Tables (1~7)

Descriptions for Supplementary Tables associated with this article:

Supplementary Table 1:

Supplementary Table 1.1: Whole-rock Zinc isotopic compositions of OIB samples from Cook-Austral Islands, St.Helena Island and Louisville seamounts

Supplementary Table 1.2: Whole-rock Zinc isotopic compositions of Archean altered oceanic crust samples in this study

Supplementary Table 1.3: Whole-rock Zinc isotopic compositions of Precambrian carbonates samples in this study

Supplementary Table 2:

Supplementary Table 2: Comparison between Zinc isotopic compositions of standards/geological reference materials in this study and published values.

Supplementary Table 3:

Modeling calculations of Zinc (Zn) isotopic variations during crystal fractionation

Supplementary Table 3.1: Variations of MgO contents during crystal fractionation

Supplementary Table 3.2: Variations of Zn isotopic values ($\delta^{66}Zn_{primary\ melt}$=0.29) during crystal fractionation

Supplementary Table 3.3: Variations of Zn isotopic values ($\delta^{66}Zn_{primary\ melt}$=0.33) during crystal fractionation

Supplementary Table 3.4: Variations of Zn isotopic values ($\delta^{66}Zn_{primary\ melt}$=0.37) during crystal fractionation

Supplementary Table 4:

The calculation for geochemical compositions of refertilized peridotite

Supplementary Table 5:

Modeling calculations of Zinc (Zn) isotopic variations during mantle partial melting.

Supplementary Table 5.1: Melting of pyroxenite for Zn isotopic compositions with evolving modal composition in melting residue

Supplementary Table 5.2: Melting of peridotite for Zn isotopic compositions with evolving modal composition in melting residue

Supplementary Table 5.3: Melting of refertilized peridotite for Zn isotopic compositions with evolving modal composition in melting residue

Supplementary Table 5.4: Melting of carbonated peridotite for Zn isotopic compositions with evolving modal composition in melting residue

Supplementary Table 6:

Modeling calculations of La/Sm variations during mantle partial melting.

Supplementary Table 6.1: Melting of pyroxenite for La/Sm with evolving modal composition in melting residue

Supplementary Table 6.2: Melting of peridotite for La/Sm with evolving modal composition in melting residue

Supplementary Table 6.3: Melting of refertilized peridotite for La/Sm with evolving modal composition in melting residue

Supplementary Table 6.4: Melting of carbonated peridotite for La/Sm with evolving modal composition in melting residue

Supplementary Table 7

Major elements, trace elements and radiogenic isotopic compositions of OIB samples from Cook-Austral Islands, St.Helena Island and Louisville seamounts.

Note 1: Description for ocean island basalt samples in this study

Geological Settings

The Cook-Austral volcanic chains, located in the South Pacific, are composed of eleven islands, two atolls and numerous seamounts. They include three hotspot tracks: (1) the Rarotonga track consisting of two main islands of Aitutaki and Rarotonga, (2) the Atiu track consisting of Atiu, Mauke and young Rurutu, and (3) the Macdonald track. The Macdonald track consists of Mangaia (19Ma, ref.[15]), Old Rurutu (12Ma, ref.[15]), Tubuai (10Ma, ref.[2]) and Raivavae (the age of Rairua-stage ranges from 7.4–10.6Ma, the ages of Anatonu-stage islands are in the range of 5.4–6.4Ma, ref.[16]). Basalts from Mangaia, old Rurutu, Tubuai, and Rairua-stage of Raivavae exhibit typical HIMU-type radiogenic isotopic compositions[2,17]. We have chosen thirteen typical HIMU OIB samples ($^{206}Pb/^{204}Pb>20.5$) from these four islands (Mangaia, old Rurutu, Tubuai, and Rairua-stage of Raivavae) and two samples with less radiogenic Pb isotopes ($^{206}Pb/^{204}Pb=19.3$ and 19.7) from Anatonu-stage of Raivavae Island.

St. Helena Island lies in the South Atlantic Ocean, consisting of two main basaltic shield volcanoes[18]. We have analyzed two samples from the northeast volcano and six samples from the southwest volcano. Three of the samples from the southwest volcano are collected on the lower shield, while two of them are from the main shield and one is from late-stage intrusions. All samples from St. Helena Island are classic HIMU OIBs ($^{206}Pb/^{204}Pb>20.5$).

The Louisville Seamounts Chain lies in the southern Pacific and is thought to have been formed by a deeply-rooted mantle plume, which originates at the core-mantle boundary[19]. Because of their moderately radiogenic Sr, Nd and Pb isotopes and primordial noble gas signature[5,20-22], the Louisville basalts have been inferred to capture a “FOZO (FOcus ZOne)” component[23], which is commonly considered to be a comparatively “primitive” component in the lower mantle without certain evidence for contributions from recycled crustal materials[23]. Louisville samples that we have analyzed were drilled at IODP site U1372 at the Canopus seamount and IODP site U1376 at Burton seamount[4,5].

Sample description

All OIB samples of this study are devoid of secondary carbonates, showing no significant chemical weathering[2,3,13]. The samples are classified as alkaline basalts (Supplementary Figure 5a),

showing low SiO_2 (41.34–46.9wt.%) and high CaO (8.56–13.59wt.%) contents (Supplementary Figure 5a and Supplementary Figure 5b), as well as high CaO/Al_2O_3 ratios (most of them are >0.8). MgO contents of the HIMU samples correlate negatively with TiO_2 and positively with CaO/Al_2O_3 ratios (Supplementary Figure 5c and Supplementary Figure 5d). In primitive-mantle-normalized, multi-incompatible-element diagrams, the HIMU samples display negative anomalies for K, Pb, Zr and Hf (Supplementary Figure 6). All HIMU samples have characteristic HIMU endmember isotopic signatures (Supplementary Figure 7a-d), with extremely radiogenic Pb ($^{206}Pb/^{204}Pb$=20.6–21.7), moderate radiogenic Nd (0.51283–0.51292) and Hf (0.28287–0.28298) isotopic compositions and unradiogenic Sr ($^{87}Sr/^{86}Sr$=0.70278–0.70290)[2,13]. Compared to classic HIMU lavas, the Pb isotopic values of our two samples from the Anatonu-stage of Raivavae Island are less radiogenic ($^{206}Pb/^{204}Pb$=19.3 and 19.7)[3]. The Louisville basalts display FOZO-type isotopic compositions thought to be representative of much of the lower mantle[23] and are included here to represent examples of isotopically "normal" OIBs. They show SiO_2 contents in the range of 43.39–44.85wt.% and high MgO contents (14.97–19.33wt.%). Detailed descriptions of these basalts can be found elsewhere[2-4,13].

Note 2: Description for ancient carbonates and altered oceanic crust samples in this study

2.1 Archean and early Proterozoic carbonates

We measured Zn isotopic composition for three Precambrian carbonate samples in this study (Supplementary Figure 8). RT35 belongs to shallow-marine carbonate, collected from ca. 2.7Ga Tumbiana Formation, Pilbara Craton. This sample is characterized by the presence of stromatolitic structure, intercalated with clastic sediments. We analyzed Zn isotopic compositions of two different parts of this sample, and obtained different δ^{66}Zn values of 0.30 ± 0.02‰ (for RT35-1, see Supplementary Figure 8) and 0.44 ± 0.01‰ (for RT35-2, see Supplementary Figure 8), respectively. TC100 and TC236 are both dolomites (Supplementary Figure 8), collected from ca. 2.3 Ga Kazput Formation, western Australia. The two samples are pure carbonates without entrained clastic components. TC100 has a δ^{66}Zn value of 0.26 ± 0.01‰, while TC236 has obviously heavier Zn isotopic composition (δ^{66}Zn=0.48 ± 0.01‰) than TC100 (Supplementary Table 1.3). Further details for these samples can be found in Komiya et al. [24].

2.2 Archean altered oceanic crust

We also analyzed Zn isotopes for sixteen Archean altered oceanic crust samples. All these samples were collected from the Salgash Subgroup (it belongs to the upper part of Warrawoona Group), eastern Pilbara Craton. Previous zircon U-Pb dating gave an age of 3.46Ga for these samples [25-27]. According to the field occurrences and microscopic characteristics, altered oceanic crust samples presented here have undergone varying degrees of hydrothermal alteration, including carbonatization and silicification. Whole-rock chemical compositions of these altered basalt samples show higher mobile element contents (e.g., K, Rb and Ba) than those of modern mid-ocean ridge basalt (MORB). Zn contents and Zn isotopic compositions of these samples vary from 38–228ppm and 0.22‰–0.49‰, respectively.

Furthermore, these altered basalts can be divided into type I and type II. Among all samples measured in this study, five of them belong to type I basalt. Such samples preserve their original igneous rock textures, showing the hyalo-ophitic and intersertal textures. The primary minerals are completely replaced by secondary minerals. The alteration mineral assemblages of type I basalt are chlorite + K-mica + quartz + carbonate minerals ± albite. Carbonate minerals here are mainly

calcite. Parts of carbonate minerals are Fe-rich, including ankerite and siderite. The whole-rock major element compositions of type I basalt show a wider range than those of modern MORB. SiO_2 and MgO contents of these samples vary from 49.95–65.37wt.% and 4.88–9.99wt.%, respectively. CaO contents are also heterogeneous with a range of 0.14–8.88wt.%. The other eleven altered samples belong to type II basalts. Due to strong hydrothermal alteration, such samples are lacking primary igneous rock textures compared to type I basalt. The alteration minerals of type II basalts are the same as in type I basalt, which consists of chlorite, K-mica, quartz, and carbonate minerals. It's worth noting that a significant number of veinlets and common pyrite (or goethite pseudomorph) exist in these samples. Most type II basalts show lower SiO_2 contents (30.77–63.68wt.%) but higher CaO contents (0.83–40.15wt.%) than type I basalt. Detailed information for these samples can be found in Nakamura and Kato [27].

Note 3: Description for quantitative modeling of zinc isotopic variations caused by crystal fractionation

Kawabata et al. [13] assumed that the primary melt of St. Helena samples has 14.6wt% MgO content. Moreover, the primary melt of St. Helena samples has experienced olivine (Ol) and clinopyroxene (Cpx) fractionation when MgO contents of magma are higher than 8wt.%. Plagioclase fractionation occurs in magma only with MgO≤8 wt.%. Magnesium contents of most St. Helena samples in this study are higher than 8wt.%, so we focus on the assessment of the effect by Ol and Cpx fractionation on whole-rock Zn isotopes. The crystal fractionation process of St. Helena magma with MgO≥8 wt.% can be divided into two-stage: Stage 1 is Ol fractionation and Stage 2 is Ol and Cpx fractionation in the proportion of 3:2 [13].

Firstly, here we assess the variation of MgO contents during crystal fractionation (Supplementary Table 3). We set $C_{\text{Mg-parental liquids}}$ as the contents of Mg in the parental liquids, $C_{\text{Mg-liquids}}$ as the contents of Mg in the liquids which is produced when parental liquids experience crystal fractionation and $C_{\text{MgO-liquids}}$ as the MgO contents in the liquids. F_1 and F_2 represent melt fractions remaining in stage 1 and stage 2, respectively. $D_{\text{Mg}}^{\text{Ol}}$ is the partition coefficient of Ol. $D_{\text{Mg}}^{\text{CPX}}$ is the partition coefficient of Cpx. $D_{\text{Mg}}^{\text{Ol:CPX=3:2}}$ is the partition coefficient of Ol and Cpx in the proportion of 3:2. $D_{\text{Mg}}^{\text{Ol}}$ and $D_{\text{Mg}}^{\text{CPX}}$ are summarized in Supplementary Information, Note 4.3.

$D_{\text{Mg}}^{\text{Ol:CPX=3:2}}$ can be calculated by

$$D_{\text{Mg}}^{\text{Ol:CPX=3:2}} = 0.6 * D_{\text{Mg}}^{\text{Ol}} + 0.4 * D_{\text{Mg}}^{\text{CPX}} \quad (1)$$

$C_{\text{Mg-parental liquids}}$ can be modeled by

$$C_{\text{Mg-parental liquids}} = C_{\text{MgO-liquids(previous step)}} \quad (2)$$

$C_{\text{Mg-liquids}}$ can be calculated by

$$C_{\text{Mg-liquids}} = C_{\text{Mg-parental liquids}} * F_1^{(D_{\text{Mg}}^{\text{Ol}}-1)}$$

or

$$C_{\text{Mg-liquids}} = C_{\text{Mg-parental liquids}} * F_1^{(D_{\text{Mg}}^{\text{Ol:CPX=3:2}}-1)} \quad (3)$$

$C_{\text{MgO-liquids}}$ can be modeled by

$$C_{\text{MgO-liquids}} = \frac{C_{\text{Mg-liquids}}}{0.6} \quad (4)$$

Subsequently, we follow the approach of Prytulak et al. [28] to evaluate the effect of crystal fractionation on Zn isotopic compositions. We set $C_{\text{Zn-parental liquids}}$ as the contents of Zn in the

parental liquids, $C_{\text{Zn-liquids}}$ as the contents of Zn in the liquids which is produced when parental liquids experience crystal fractionation. All these factors can be modeled as equation (2) and (3). $D_{\text{Zn}}^{\text{Ol}}$ is the partition coefficient of Ol. $D_{\text{Zn}}^{\text{CPX}}$ is the partition coefficient of Cpx. $D_{\text{Zn}}^{\text{Ol:CPX=3:2}}$is the partition coefficient of Ol and Cpx in the proportion of 3:2. $D_{\text{Zn}}^{\text{Ol}}$ and $D_{\text{Zn}}^{\text{CPX}}$are summarized in Supplementary Information, Note 4.3. $D_{\text{Zn}}^{\text{Ol:CPX=3:2}}$ can be calculated as equation (1).

F_1 and F_2 represent melt fractions remaining in stage 1 and stage 2, respectively. f_1 and f_2 represent the fraction of Zn remaining in the liquid in stage 1 and stage 2, respectively. f_1 and f_2 can be modeled by

$$f_1 = \frac{F_1}{F_1 + D_{\text{Zn}}^{\text{Ol}} * (1 - F_1)}$$

or

$$f_2 = \frac{F_2}{F_2 + D_{\text{Zn}}^{\text{Ol:CPX=3:2}} * (1 - F_2)} \quad (5)$$

$\delta^{66}\text{Zn}_{\text{parental-liquids}}$ is $\delta^{66}\text{Zn}$ values of parental liquids and $\delta^{66}\text{Zn}_{\text{liquids}}$ is $\delta^{66}\text{Zn}$ values of liquids which is produced when parental liquids experience crystal fractionation.

$\delta^{66}\text{Zn}_{\text{parental-liquids}}$ can be calculated by

$$\delta^{66}\text{Zn}_{\text{parental-liquids}} = \delta^{66}\text{Zn}_{\text{liquids(previous step)}} \quad (6)$$

$\delta^{66}\text{Zn}_{\text{liquids}}$ can be modeled by

$$\delta^{66}\text{Zn}_{\text{liquids}} = \delta^{66}\text{Zn}_{\text{parental-liquids}} + 1000 * \text{LN}\alpha_{\text{Zn}}^{\text{Ol}} * \text{LN}f_1$$

or

$$\delta^{66}\text{Zn}_{\text{liquids}} = \delta^{66}\text{Zn}_{\text{parental-liquids}} + 1000 * \text{LN}\alpha_{\text{Zn}}^{\text{Ol:cpx=3:2}} * \text{LN}f_2 \quad (7)$$

where $\alpha_{\text{Zn}}^{\text{Ol}}$ and $\alpha_{\text{Zn}}^{\text{Ol:cpx=3:2}}$ represent the fractionation factor between minerals and liquids.

Note 4: Description for quantitative modeling of partial melting of different mantle lithologies

4.1 Calculation for partial melting

We follow the approach of Williams and Bizimis [29] and Zhong et al. [4] to perform the modeling of partial melting of different mantle lithologies in this study (Supplementary Table 5 and Supplementary Table 6). We use an incremental batch melting model to evaluate the effect of the partial melting process on the Zn isotopic fractionation. Clinopyroxene (Cpx) and garnet in peridotite were modeled to be exhausted at around 30% degree of melting.

We set F_0 as melt increment to represent each step of mass melted and each step is constantly set to 1% of the bulk mass. f_n represents the melting degree for the residue of each step n. f_n can be calculated by

$$f_n = \frac{F_0}{1-(n-1)\,F_0} \tag{8}$$

The Zn contents in the melt and residue can be denoted by c_n^{melt} and c_n^{residue}, respectively, so we can obtain Zn contents by

$$c_n^{\mathrm{melt}} = \frac{c_{n-1}^{\mathrm{residue}}}{D_n + (1-P)\,f_n} \tag{9}$$

$$c_n^{\mathrm{residue}} = \frac{c_{n-1}^{\mathrm{residue}} - c_n^{\mathrm{melt}} * f_n}{1-f_n} \tag{10}$$

Where D_n is the bulk partition coefficient and P is the mineral proportional partition coefficient.

$$D_n^{\mathrm{Zn}} = \sum \mathrm{Initial}\ Mode_{\mathrm{mineral}} * Kd_{\mathrm{Zn}}^{\mathrm{mineral/melt}} \tag{11}$$

$$P_n^{\mathrm{Zn}} = \sum \mathrm{Melting}\ Mode_{\mathrm{mineral}} * Kd_{\mathrm{Zn}}^{\mathrm{mineral/melt}} \tag{12}$$

According to Williams and Bizimis [29] and Zhong et al. [4], here we treat $\alpha_n^{\mathrm{melt-residue}}$ as the fractionation factor between the melt and residue. It can be modeled by

$$\alpha_n^{\mathrm{melt-residue}} = \alpha_{\mathrm{melt\text{-}cpx}} * \left(\frac{\sum_{i-1}^{n}[n_i * \mathrm{Zn}_{\mathrm{mineral}}]}{\sum_{i=1}^{n}[n_i * \mathrm{Zn}_{\mathrm{mineral}} * \alpha_{\mathrm{minerel-cpx}}]} \right) \tag{13}$$

Based on the values of $\alpha_n^{\mathrm{melt-residue}}$ calculated by equation (13), we, therefore, can use equation (14) to get $\Delta_n^{\mathrm{melt-residue}}$ values, which represents the fractionation of Zn isotopes between melt and residue.

$$\Delta_n^{\mathrm{melt-residue}} = 1000\mathrm{LN}\alpha_n^{\mathrm{melt-residue}} \tag{14}$$

Therefore, zinc isotopic values of instantaneous melts can be represented by $\delta^{66}\mathrm{Zn}_n^{\mathrm{inst.melt}}$.

When n is equal to 1, it can be modeled by

$$\delta^{66}\mathrm{Zn}_n^{\mathrm{inst.melt}} = \frac{\delta^{66}\mathrm{Zn}_0 * c_0 + \Delta_n^{\mathrm{melt-residue}} * c_n^{\mathrm{residue}} * (1-f_n)}{c_n^{\mathrm{melt}} * f_n + c_n^{\mathrm{residue}} * (1-f_n)} \quad (15)$$

Where $\delta^{66}\mathrm{Zn}_0$ is initial $\delta^{66}\mathrm{Zn}$ value and c_0 is initial bulk Zn content.

When n is greater than 1, $\delta^{66}\mathrm{Zn}_n^{\mathrm{inst.melt}}$ can be calculated by

$$\delta^{66}\mathrm{Zn}_n^{\mathrm{inst.melt}} = \frac{\delta^{66}\mathrm{Zn}_{n-1}^{\mathrm{residue}} * c_{n-1}^{\mathrm{residue}} + \Delta_n^{\mathrm{melt-residue}} * c_n^{\mathrm{residue}} * (1-f_n)}{c_n^{\mathrm{melt}} * f_n + c_n^{\mathrm{residue}} * (1-f_n)} \quad (16)$$

In equation (16), $\delta^{66}Zn_n^{\mathrm{residue}}$ can be calculated by

$$\delta^{66}\mathrm{Zn}_n^{\mathrm{residue}} = \delta^{66}\mathrm{Zn}_n^{\mathrm{inst.melt}} - \Delta_n^{\mathrm{melt-residue}} \quad (17)$$

Zn isotopic values of aggregated melts can be represented by $\delta^{66}\mathrm{Zn}_n^{\mathrm{aggr.melt}}$

When n is equal to 1,

$$\delta^{66}\mathrm{Zn}_n^{\mathrm{aggr.melt}} = \delta^{66}\mathrm{Zn}_n^{\mathrm{inst.melt}} \quad (18)$$

When n is greater than 1, $\delta^{66}\mathrm{Zn}_n^{\mathrm{aggr.melt}}$ can be calculated by

$$\delta^{66}\mathrm{Zn}_n^{\mathrm{aggr.melt}} = \frac{\sum_{i=1}^{n}[c_i^{\mathrm{melt}} * \delta^{66}\mathrm{Zn}_i^{\mathrm{inst.melt}}]}{\sum_{i=1}^{n}[c_n^{\mathrm{melt}}]} \quad (19)$$

4.2 Calculation for the geochemical composition of refertilized peridotite

Herzberg et al. [30] proposed a refertilized peridotite source in the HIMU source. Such refertilized peridotite can be generated in the following way: subducted mafic crust release silicic melts to metasomatize the surrounding peridotite, and therefore generate such refertilized peridotite. Taking Herzberg et al. [30]'s model into account, we calculate the initial geochemical compositions (initial Zn, La and Sm contents as well as δ^{66}Zn) of such refertilized peridotite in this part (Supplementary Information, Note 4.2).

The chemical composition (Zn, La and Sm contents as well as δ^{66}Zn) of silicic melt derived from recycled mafic crust can be assumed by melts of pyroxenite with 10% degree of partial melting. The detailed calculation for melting of pyroxenite is given in Supplementary Information, Note 4.1, and calculated results can be found in Supplementary Table 4, Supplementary Table 5.1 and Supplementary Table 6.1. Herzberg et al. [30] infer that normal peridotite has been fertilized by 10% addition of silicic melts released from pyroxenite components, yielding such refertilized peridotite. Therefore, initial Zn, La and Sm contents of refertilized peridotite can be modeled by

$$c_{\mathrm{Zn}}^{\mathrm{refertilized\ peridotite}} = 0.1 * c_{\mathrm{Zn}}^{\mathrm{silicic\ melt}} + 0.9 * c_{\mathrm{Zn}}^{\mathrm{peridotite}} \quad (20)$$

$$c_{\mathrm{La}}^{\mathrm{refertilized\ peridotite}} = 0.1 * c_{\mathrm{La}}^{\mathrm{silicic\ melt}} + 0.9 * c_{\mathrm{La}}^{\mathrm{peridotite}} \quad (21)$$

$$c_{\mathrm{Sm}}^{\mathrm{refertilized\ peridotite}} = 0.1 * c_{\mathrm{Sm}}^{\mathrm{silicic\ melt}} + 0.9 * c_{\mathrm{Sm}}^{\mathrm{peridotite}} \quad (22)$$

Where $c_{\text{Zn}}^{\text{peridotite}}$, $c_{\text{La}}^{\text{peridotite}}$ and $c_{\text{Sm}}^{\text{peridotite}}$ are initial Zn, La and Sm contents of normal peridotite and are given in Supplementary Information, Note 4.3.

Moreover, $\delta^{66}\text{Zn}_0^{\text{refertilized peridotite}}$ is initial δ^{66}Zn values of refertilized peridotite and can be calculated by

$$\delta^{66}\text{Zn}_0^{\text{refertilized peridotite}} = \frac{\delta^{66}\text{Zn}^{\text{silicic melt}} * 0.1 * c_{\text{Zn}}^{\text{silicic melt}}}{c_{\text{Zn}}^{\text{refertilized peridotite}}} + \frac{\delta^{66}\text{Zn}^{\text{peridotite}} * 0.9 * c_{\text{Zn}}^{\text{peridotite}}}{c_{\text{Zn}}^{\text{refertilized peridotite}}} \quad (23)$$

Furthermore, the calculation for partial melting of such refertilized peridotite is the same as that described in Supplementary Information, Note 4.1. and calculated results for partial melting of such refertilized peridotite can be found in Supplementary Table 5.3 and Supplementary Table 6.3.

4.3 Model input

4.3.1 The model input of modeling calculation for Zn isotope

The initial mineral modal abundance and melting mode for pyroxenite are from Williams and Bizimis [29]. The initial mineral modal abundance and melting mode for peridotite and refertilized peridotite are taken from Walter [31]. The initial mineral modal abundance and melting mode for carbonated peridotite are from Zeng et al. [32]. Initial δ^{66}Zn values of pyroxenite are equal to 0.27‰ and taken from Huang et al. [33], Inglis et al. [10] and Wang et al. [6]. Initial δ^{66}Zn values ($\delta^{66}\text{Zn}_0$) of peridotite is equal to 0.16‰ and taken from Sossi et al. [1]. Initial δ^{66}Zn values ($\delta^{66}\text{Zn}_0$) of refertilized peridotite is equal to 0.19‰ and calculated in Supplementary Information, Note 4.2. We assumed the initial Zn isotopic composition of carbonated peridotite as 0.30‰. $Kd_{\text{Zn}}^{\text{mineral/melt}}$ values and initial Zn contents for pyroxenite are from Pertermann et al. [34]. $Kd_{\text{Zn}}^{\text{mineral/melt}}$ values for peridotite and refertilized peridotite are from Davis et al. [35]. $Kd_{\text{Zn}}^{\text{mineral/melt}}$ values for carbonated peridotite are from Adam and Green [36]. Initial Zn content for pyroxenite is assumed by the average of all minerals. Initial Zn contents for peridotite are calculated from Le Roux et al. [37] and Wang et al. [6]. Initial Zn contents for refertilized peridotite are calculated in Supplementary Information, Note 4.2. Initial Zn contents (bulk Zn) for carbonated peridotite are from Le Roux et al. [37] and Wang et al. [6]. Previous studies have suggested that the degree of Zn isotope fractionation induced by melting in the garnet stability field model is very similar to that estimated for melting in the spinel stability field[1,38]. Observations on natural samples also support this point. The other OIBs produced by melting of CO_2-free garnet peridotitic mantle have indistinguishable Zn isotopic compositions from MORB that produced by melting of spinel peridotite (Supplementary Figure 1). Thus, melting of

garnet- and spinel-facies mantle has similar effect on Zn isotopic fractionation [1,6,38], and it is reasonable to use α (including $\alpha_{\text{melt-cpx}}$, $\alpha_{\text{ol-cpx}}$, $\alpha_{\text{opx-cpx}}$ and $\alpha_{\text{grt-cpx}}$) given in McCoy-West et al. [38], Sossi et al. [1] and Wang et al. [6].

4.3.2 The model input of modeling calculation for La/Sm ratio

The initial mineral modal abundance and melting mode for pyroxenite are from Williams and Bizimis [29]. The initial mineral modal abundance and melting mode for peridotite and refertilized peridotite are taken from Walter [31]. The initial mineral modal abundance and melting mode for carbonated peridotite are from Zeng et al. [32]. $Kd_{\text{La}}^{\text{mineral/melt}}$ and $Kd_{\text{Sm}}^{\text{mineral/melt}}$ values for pyroxenite are from Pertermann and Hirschmann [39]. $Kd_{\text{La}}^{\text{mineral/melt}}$ and $Kd_{\text{Sm}}^{\text{mineral/melt}}$ values for peridotite and refertilized peridotite are from Green et al. [40]. $Kd_{\text{La}}^{\text{mineral/melt}}$ and $Kd_{\text{Sm}}^{\text{mineral/melt}}$ values for carbonated peridotite are taken from Zanetti et al. [41] and Fujimaki et al. [42]. Initial La and Sm contents of pyroxenites are represented by those of average MORB from Gale et al. [43]. Initial La and Sm contents of peridotite are represented by primitive mantle values from McDonough and Sun [44]. Initial La and Sm contents of refertilized peridotite are calculated in Supplementary Information, Note 4.2. For La and Sm contents of carbonated peridotite, we use calculated results by mixing of 0.2% calcio-carbonatite with primitive mantle values[44]. The La and Sm concentrations of carbonatite are taken from Hoernle et al. [45].

Note 5: Description for quantitative modeling of mass balance for zinc isotopes

In this study, we take a quantitative modeling to evaluate how much recycled carbonates are required to form a HIMU source with $\delta^{66}Zn$ values of 0.30‰, following the approach of Wang et al. [46]. Such approach can be simplified by a three-component mixture model as that shows in ref.[46]. These three components are mantle peridotite, ancient subducted sediments and silicic melt released from recycled crust, respectively. Initial Zn content of mantle peridotite can be calculated by data in ref.[6,37]. Initial $\delta^{66}Zn$ value of mantle peridotite is from ref.[1]. Initial Zn content of silicic melt released from recycled crust is equal to 69ppm. Initial $\delta^{66}Zn$ value of silicic melt is equal to 0.31‰. Detailed calculation for composition of such silicic melt can be found in Supplementary Information, Note 4.1. Ancient subducted sediments are composed by 40% of silicate sediments and 60% of carbonates. Initial Zn content and $\delta^{66}Zn$ value of silicate sediments are averaged by data from ref.[47-50]. Initial Zn content and $\delta^{66}Zn$ value of carbonates are averaged by data from refs. [50-56].

Note 6: Data source for Figure 1, Figure 3 and Figure 4

Data source for Figure 1:

Data for peridotite massifs/xenoliths are from ref.[1,6]. Data for altered oceanic crust consist of data in this study and literature data [10,33]. Here we analyzed Zn isotopes of sixteen Archean (3.46Ga) altered oceanic crust samples, and their $\delta^{66}Zn$ values vary from 0.22‰ to 0.49‰. Further details and $\delta^{66}Zn$ values for these Archean altered oceanic crust can be found in Supplementary information, Note 2 and Supplementary Table 1.2. Data for MORB (Mid-ocean ridge basalt) are from ref. [6,37]. Data for marine sediments are summarized from ref.[50]. Data for Precambrian carbonates include data from this study and reference data from ref.[52-55]. Further details and isotopic values for these Precambrian carbonates are given in Supplementary information, Note 2 and Supplementary Table 1.3. Data for Phanerozoic carbonates are given by ref.[50,51,57-63] and reference therein.

Data source for Figure 3a:

Average Os isotopic composition of MORB and primitive upper mantle (PUM) are calculated from ref.[64] and ref.[65], respectively. Average Zn isotopic composition of MORB is calculated from ref.[6,9]. The $\delta^{66}Zn$ values of PUM is from ref.[1].

Data source for Figure 3b:

$^{87}Sr/^{86}Sr$ value of ambient peridotite in 2.45Ga ago is calculated by present values of $^{87}Sr/^{86}Sr=0.70263$ and $^{87}Rb/^{86}Sr=0.0065$ [66]. $^{87}Sr/^{86}Sr$ value of carbonatite melt inherits from recycled crustal materials, including altered oceanic crust and carbonate-bearing sediment. $^{87}Sr/^{86}Sr$ value of altered oceanic crust in 2.45Ga ago is based on present values of $^{87}Sr/^{86}Sr=0.702962$ and $^{87}Rb/^{86}Sr=0.051$ [67]. $^{87}Sr/^{86}Sr$ value of carbonate-bearing sediment in 2.45Ga ago is based on present values of $^{87}Sr/^{86}Sr=0.72$(ref.[68]), $^{87}Sr/^{86}Sr=0.704551$(refs.[69,70]), $^{87}Rb/^{86}Sr=0.495$(ref.[68])and $^{87}Rb/^{86}Sr=0.005$ (ref. [69]). The metasomatized event is assumed to occur at 2.45Ga [71]. Rubidium (Rb) and Strontium (Sr) contents of carbonatite melt are from Walter et al. [72].

Data source for Figure 4:

The average value for MORB is calculated by data from ref.[6,37]. The average value for altered oceanic crust is calculated by data measured in this study and data from ref. [10,33]. The average value for sedimentary carbonates is calculated by data measured in this study and data from ref.[50-55,57-63].

Supplementary References

1. Sossi, P. A., Nebel, O., O'Neill, H. S. C. & Moynier, F. Zinc isotope composition of the Earth and its behaviour during planetary accretion. *Chem. Geol.* **477**, 73-84 (2018).
2. Hanyu, T., et al. Geochemical characteristics and origin of the HIMU reservoir: A possible mantle plume source in the lower mantle. *Geochem. Geophys. Geosyst.* **12**, Q0AC09 (2011).
3. Miyazaki, T., et al. Clinopyroxene and bulk rock Sr–Nd–Hf–Pb isotope compositions of Raivavae ocean island basalts: Does clinopyroxene record early stage magma chamber processes? *Chem. Geol.* **482**, 18-31 (2018).
4. Zhong, Y., et al. Magnesium isotopic variation of oceanic island basalts generated by partial melting and crustal recycling. *Earth. Planet. Sci. Lett.* **463**, 127-135 (2017).
5. Shi, J.-H., et al. An eclogitic component in the Pitcairn mantle plume: Evidence from olivine compositions and Fe isotopes of basalts. *Geochim. Cosmochim. Acta.* **318**, 415-427 (2022).
6. Wang, Z.-Z., et al. Zinc isotope fractionation during mantle melting and constraints on the Zn isotope composition of Earth's upper mantle. *Geochim. Cosmochim. Acta.* **198**, 151-167 (2017).
7. Chen, H., Savage, P. S., Teng, F.-Z., Helz, R. T. & Moynier, F. Zinc isotope fractionation during magmatic differentiation and the isotopic composition of the bulk Earth. *Earth. Planet. Sci. Lett.* **369-370**, 34-42 (2013).
8. Yao, J., Huang, J. & Zhang, G. Zinc isotope constraints on carbonated mantle sources for rejuvenated-stage lavas from Kaua'i, Hawai'i. *Chem. Geol.* **605**, 120967 (2022).
9. Huang, J., et al. Zinc isotopic systematics of Kamchatka-Aleutian arc magmas controlled by mantle melting. *Geochim. Cosmochim. Acta.* **238**, 85-101 (2018).
10. Inglis, E. C., et al. The behavior of iron and zinc stable isotopes accompanying the subduction of mafic oceanic crust: A case study from Western Alpine ophiolites. *Geochem. Geophys. Geosyst.* **18**, 2562-2579 (2017).
11. Zeng, G., et al. Nephelinites in eastern China originating from the mantle transition zone. *Chem. Geol.* **576**, 120276 (2021).
12. Wang, Z.-Z., Liu, S.-A., Chen, L.-H., Li, S.-G. & Zeng, G. Compositional transition in natural alkaline lavas through silica-undersaturated melt–lithosphere interaction. *Geology.* **46**, 771-774 (2018).
13. Kawabata, H., et al. The Petrology and Geochemistry of St. Helena Alkali Basalts: Evaluation of the Oceanic Crust-recycling Model for HIMU OIB. *J. Petrol.* **52**, 791-838 (2011).
14. Vervoort, J. D., Plank, T. & Prytulak, J. The Hf–Nd isotopic composition of marine sediments. *Geochim. Cosmochim. Acta.* **75**, 5903-5926 (2011).
15. Bonneville, A., Dosso, L. & Hildenbrand, A. Temporal evolution and geochemical variability of the South Pacific superplume activity. *Earth. Planet. Sci. Lett.* **244**, 251-269 (2006).
16. Maury, R. C., et al. Temporal evolution of a Polynesian hotspot: New evidence from Raivavae (Austral islands, South Pacific ocean). *Bull. Soc. Geol. Fr.* **184**, 557-567 (2013).
17. Chauvel, C., McDonough, W., Guille, G., Maury, R. & Duncan, R. Contrasting old and young volcanism in Rurutu Island, Austral chain. *Chem. Geol.* **139**, 125-143 (1997).
18. Baker, I., Gale, N. H. & Simons, J. Geochronology of the St Helena Volcanoes. *Nature.* **215**, 1451-1456 (1967).
19. Courtillot, V., Davaille, A., Besse, J. & Stock, J. Three distinct types of hotspots in the Earth's mantle. *Earth. Planet. Sci. Lett.* **205**, 295-308 (2003).

20. Hanyu, T. Deep plume origin of the Louisville hotspot: Noble gas evidence. *Geochem. Geophys. Geosyst.* **15**, 565-576 (2014).

21. Stracke, A., Hofmann, A. W. & Hart, S. R. FOZO, HIMU, and the rest of the mantle zoo. *Geochem. Geophys. Geosyst.* **6**, Q05007 (2005).

22. Vanderkluysen, L., et al. Louisville Seamount Chain: Petrogenetic processes and geochemical evolution of the mantle source. *Geochem. Geophys. Geosyst.* **15**, 2380-2400 (2014).

23. Hart, S. R., Hauri, E. H., Oschmann, L. A. & Whitehead, J. A. Mantle Plumes and Entrainment: Isotopic Evidence. *Science.* **256**, 517-520 (1992).

24. Komiya, T., et al. Evolution of the composition of seawater through geologic time, and its influence on the evolution of life. *Gondwana. Res.* **14**, 159-174 (2008).

25. Thorpe, R. I., Hickman, A. H., Davis, D. W., Mortensen, J. K. & Trendall, A. F. U-Pb zircon geochronology of Archaean felsic units in the Marble Bar region, Pilbara Craton, Western Australia. *Precambrian Res.* **56**, 169-189 (1992).

26. McNaughton, N. J., Compston, W. & Barley, M. E. Constraints on the age of the Warrawoona Group, eastern Pilbara Block, Western Australia. *Precambrian Res.* **60**, 69-98 (1993).

27. Nakamura, K. & Kato, Y. Carbonatization of oceanic crust by the seafloor hydrothermal activity and its significance as a CO2 sink in the Early Archean1. *Geochim. Cosmochim. Acta.* **68**, 4595-4618 (2004).

28. Prytulak, J., et al. Stable vanadium isotopes as a redox proxy in magmatic systems? *Geochem. Perspect. Lett.* **3**, 75-84 (2016).

29. Williams, H. M. & Bizimis, M. Iron isotope tracing of mantle heterogeneity within the source regions of oceanic basalts. *Earth. Planet. Sci. Lett.* **404**, 396-407 (2014).

30. Herzberg, C., et al. Phantom Archean crust in Mangaia hotspot lavas and the meaning of heterogeneous mantle. *Earth. Planet. Sci. Lett.* **396**, 97-106 (2014).

31. Walter, M. J. Melting of Garnet Peridotite and the Origin of Komatiite and Depleted Lithosphere. *J. Petrol.* **39**, 29-60 (1998).

32. Zeng, G., Chen, L.-H., Xu, X.-S., Jiang, S.-Y. & Hofmann, A. W. Carbonated mantle sources for Cenozoic intra-plate alkaline basalts in Shandong, North China. *Chem. Geol.* **273**, 35-45 (2010).

33. Huang, J., Liu, S.-A., Gao, Y., Xiao, Y. & Chen, S. Copper and zinc isotope systematics of altered oceanic crust at IODP Site 1256 in the eastern equatorial Pacific. *J. Geophys. Res.-Sol. Ea.* **121**, 7086-7100 (2016).

34. Pertermann, M., Hirschmann, M. M., Hametner, K., Günther, D. & Schmidt, M. W. Experimental determination of trace element partitioning between garnet and silica-rich liquid during anhydrous partial melting of MORB-like eclogite. *Geochem. Geophys. Geosyst.* **5**, (2004).

35. Davis, F. A., Humayun, M., Hirschmann, M. M. & Cooper, R. S. Experimentally determined mineral/melt partitioning of first-row transition elements (FRTE) during partial melting of peridotite at 3GPa. *Geochim. Cosmochim. Acta.* **104**, 232-260 (2013).

36. Adam, J. & Green, T. Trace element partitioning between mica- and amphibole-bearing garnet lherzolite and hydrous basanitic melt: 1. Experimental results and the investigation of controls on partitioning behaviour. *Contrib. Mineral. Petrol.* **152**, 1-17 (2006).

37. Le Roux, V., Lee, C. T. A. & Turner, S. J. Zn/Fe systematics in mafic and ultramafic systems: Implications for detecting major element heterogeneities in the Earth's mantle. *Geochim. Cosmochim. Acta.* **74**, 2779-2796 (2010).

38. McCoy-West, A. J., Fitton, J. G., Pons, M.-L., Inglis, E. C. & Williams, H. M. The Fe and Zn isotope

composition of deep mantle source regions: Insights from Baffin Island picrites. *Geochim. Cosmochim. Acta.* **238**, 542-562 (2018).

39. Pertermann, M. & Hirschmann, M. M. Anhydrous Partial Melting Experiments on MORB-like Eclogite: Phase Relations, Phase Compositions and Mineral–Melt Partitioning of Major Elements at 2–3 GPa. *J. Petrol.* **44**, 2173-2201 (2003).
40. Green, T. H., Blundy, J. D., Adam, J. & Yaxley, G. M. SIMS determination of trace element partition coefficients between garnet, clinopyroxene and hydrous basaltic liquids at 2–7.5 GPa and 1080–1200°C. *Lithos.* **53**, 165-187 (2000).
41. Zanetti, A., Tiepolo, M., Oberti, R. & Vannucci, R. Trace-element partitioning in olivine: modelling of a complete data set from a synthetic hydrous basanite melt. *Lithos.* **75**, 39-54 (2004).
42. Fujimaki, H., Tatsumoto, M. & Aoki, K.-i. Partition coefficients of Hf, Zr, and ree between phenocrysts and groundmasses. *J. Geophys. Res.-Sol. Ea.* **89**, B662-B672 (1984).
43. Gale, A., Dalton, C. A., Langmuir, C. H., Su, Y. & Schilling, J.-G. The mean composition of ocean ridge basalts. *Geochem. Geophys. Geosyst.* **14**, 489-518 (2013).
44. McDonough, W. F. & Sun, S. s. The composition of the Earth. *Chem. Geol.* **120**, 223-253 (1995).
45. Hoernle, K., Tilton, G., Le Bas, M. J., Duggen, S. & Garbe-Schönberg, D. Geochemistry of oceanic carbonatites compared with continental carbonatites: mantle recycling of oceanic crustal carbonate. *Contrib. Mineral. Petrol.* **142**, 520-542 (2002).
46. Wang, X.-J., et al. Recycled ancient ghost carbonate in the Pitcairn mantle plume. *Proc. Natl Acad. Sci.* **115**, 8682-8687 (2018).
47. Bentahila, Y., Ben Othman, D. & Luck, J.-M. Strontium, lead and zinc isotopes in marine cores as tracers of sedimentary provenance: A case study around Taiwan orogen. *Chem. Geol.* **248**, 62-82 (2008).
48. Maréchal, C. N., Nicolas, E., Douchet, C. & Albarède, F. Abundance of zinc isotopes as a marine biogeochemical tracer. *Geochem. Geophys. Geosyst.* **1**, 1015 (2000).
49. Andersen, M. B., et al. The Zn abundance and isotopic composition of diatom frustules, a proxy for Zn availability in ocean surface seawater. *Earth. Planet. Sci. Lett.* **301**, 137-145 (2011).
50. Pons, M.-L., et al. Early Archean serpentine mud volcanoes at Isua, Greenland, as a niche for early life. *Proc. Natl Acad. Sci.* **108**, 17639-17643 (2011).
51. Pichat, S., Douchet, C. & Albarède, F. Zinc isotope variations in deep-sea carbonates from the eastern equatorial Pacific over the last 175 ka. *Earth. Planet. Sci. Lett.* **210**, 167-178 (2003).
52. John, S. G., Kunzmann, M., Townsend, E. J. & Rosenberg, A. D. Zinc and cadmium stable isotopes in the geological record: A case study from the post-snowball Earth Nuccaleena cap dolostone. *Palaeogeogr., Palaeoclimatol., Palaeoecol.* **466**, 202-208 (2017).
53. Lv, Y., et al. Zn-Sr isotope records of the Ediacaran Doushantuo Formation in South China: diagenesis assessment and implications. *Geochim. Cosmochim. Acta.* **239**, 330-345 (2018).
54. Yan, B., Zhu, X., He, X. & Tang, S. Zn isotopic evolution in early Ediacaran ocean: A global signature. *Precambrian Res.* **320**, 472-483 (2019).
55. Kunzmann, M., et al. Zn isotope evidence for immediate resumption of primary productivity after snowball Earth. *Geology.* **41**, 27-30 (2013).
56. Li, J.-L., Klemd, R., Gao, J. & Meyer, M. Compositional zoning in dolomite from lawsonite-bearing eclogite (SW Tianshan, China): Evidence for prograde metamorphism during subduction of oceanic crust. *Am. Mineral.* **99**, 206-217 (2014).

57. Little, S. H., et al. Cold-water corals as archives of seawater Zn and Cu isotopes. *Chem. Geol.* **578**, 120304 (2021).
58. Qu, Y.-R., Liu, S.-A., Wu, H., Li, M.-L. & Tian, H.-C. Tracing carbonate dissolution in subducting sediments by zinc and magnesium isotopes. *Geochim. Cosmochim. Acta.* **319**, 56-72 (2022).
59. Zhao, M., et al. Evaluation of shallow-water carbonates as a seawater zinc isotope archive. *Earth. Planet. Sci. Lett.* **553**, 116599 (2021).
60. Sweere, T. C., et al. Isotopic evidence for changes in the zinc cycle during Oceanic Anoxic Event 2 (Late Cretaceous). *Geology.* **46**, 463-466 (2018).
61. Wang, X., Liu, S.-A., Wang, Z., Chen, D. & Zhang, L. Zinc and strontium isotope evidence for climate cooling and constraints on the Frasnian-Famennian (~372 Ma) mass extinction. *Palaeogeogr., Palaeoclimatol., Palaeoecol.* **498**, 68-82 (2018).
62. Liu, S.-A., et al. Zinc isotope evidence for intensive magmatism immediately before the end-Permian mass extinction. *Geology.* **45**, 343-346 (2017).
63. Chen, X., et al. Zinc isotope evidence for paleoenvironmental changes during Cretaceous Oceanic Anoxic Event 2. *Geology.* **49**, 412-416 (2020).
64. Gannoun, A., et al. The scale and origin of the osmium isotope variations in mid-ocean ridge basalts. *Earth. Planet. Sci. Lett.* **259**, 541-556 (2007).
65. Meisel, T., Walker, R. J. & Morgan, J. W. The osmium isotopic composition of the Earth's primitive upper mantle. *Nature.* **383**, 517-520 (1996).
66. Workman, R. K. & Hart, S. R. Major and trace element composition of the depleted MORB mantle (DMM). *Earth. Planet. Sci. Lett.* **231**, 53-72 (2005).
67. Delavault, H., Chauvel, C., Thomassot, E., Devey, C. W. & Dazas, B. Sulfur and lead isotopic evidence of relic Archean sediments in the Pitcairn mantle plume. *Proc. Natl Acad. Sci.* **113**, 12952 (2016).
68. Stracke, A., Bizimis, M. & Salters, V. J. M. Recycling oceanic crust: Quantitative constraints. *Geochemistry Geophysics Geosystems.* **4**, (2003).
69. Kamber, B. S. & Webb, G. E. The geochemistry of late Archaean microbial carbonate: implications for ocean chemistry and continental erosion history. *Geochim. Cosmochim. Acta.* **65**, 2509-2525 (2001).
70. Veizer, J., Hoefs, J., Lowe, D. R. & Thurston, P. C. Geochemistry of Precambrian carbonates: II. Archean greenstone belts and Archean sea water. *Geochim. Cosmochim. Acta.* **53**, 859-871 (1989).
71. Cabral, R. A., et al. Anomalous sulphur isotopes in plume lavas reveal deep mantle storage of Archaean crust. *Nature.* **496**, 490-493 (2013).
72. Walter, M. J., et al. Primary carbonatite melt from deeply subducted oceanic crust. *Nature.* **454**, 622-625 (2008).
