# Supplementary material for: Zinc isotopic evidence for recycled carbonate in the deep mantle
Source: Nat Commun. 2022 Oct 14;13:6085. doi: 10.1038/s41467-022-33789-6 (PMC9568527; doi:10.1038/s41467-022-33789-6)
Supplement: Supplementary file 3 — Description of Additional Supplementary files [file 41467_2022_33789_MOESM3_ESM.pdf]

Description of Additional Supplementary Files

File name: Supplementary Table 1
Description: Supplementary Table 1.1: Whole-rock Zinc isotopic compositions of OIB samples from Cook-Austral Islands, St.Helena Island and Louisville seamounts
Supplementary Table 1.2: Whole-rock Zinc isotopic compositions of Archean altered oceanic crust samples in this study
Supplementary Table 1.3: Whole-rock Zinc isotopic compositions of Precambrian carbonates samples in this study

File name: Supplementary Table 2
Description: Comparison between Zinc isotopic compositions of standards/geological reference materials in this study and published values.

File name: Supplementary Table 3
Description: Modeling calculations of Zinc (Zn) isotopic variations during crystal fractionation
Supplementary Table 3.1: Variations of MgO contents during crystal fractionation
Supplementary Table 3.2: Variations of Zn isotopic values ($\delta^{66}Zn_{primary\ melt}$=0.29) during crystal fractionation
Supplementary Table 3.3: Variations of Zn isotopic values ($\delta^{66}Zn_{primary\ melt}$=0.33) during crystal fractionation
Supplementary Table 3.4: Variations of Zn isotopic values ($\delta^{66}Zn_{primary\ melt}$=0.37) during crystal fractionation

File name: Supplementary Table 4
Description: The calculation for geochemical compositions of refertilized peridotite

File name: Supplementary Table 5
Description: Modeling calculations of Zinc (Zn) isotopic variations during mantle partial melting.
Supplementary Table 5.1: Melting of pyroxenite for Zn isotopic compositions with evolving modal composition in melting residue
Supplementary Table 5.2: Melting of peridotite for Zn isotopic compositions with evolving modal composition in melting residue
Supplementary Table 5.3: Melting of refertilized peridotite for Zn isotopic compositions with evolving modal composition in melting residue
Supplementary Table 5.4: Melting of carbonated peridotite for Zn isotopic compositions with evolving modal composition in melting residue

File name: Supplementary Table 6:
Description: Modeling calculations of La/Sm variations during mantle partial melting.
Supplementary Table 6.1: Melting of pyroxenite for La/Sm with evolving modal composition in melting residue
Supplementary Table 6.2: Melting of peridotite for La/Sm with evolving modal composition in melting residue
Supplementary Table 6.3: Melting of refertilized peridotite for La/Sm with evolving modal composition in melting residue
Supplementary Table 6.4: Melting of carbonated peridotite for La/Sm with evolving modal composition in melting residue

File name: Supplementary Table 7
Description: Major elements, trace elements and radiogenic isotopic compositions of OIB samples from Cook-Austral Islands, St.Helena Island and Louisville seamounts
